# Supplementary figures and images for: Tunneled peripherally inserted central catheter versus non-tunneled and its effects in clinical outcomes: A multicenter randomized clinical trial protocol
Source: PLoS One. 2026 Mar 10;21(3):e0342385. doi: 10.1371/journal.pone.0342385 (PMC12974855; doi:10.1371/journal.pone.0342385)

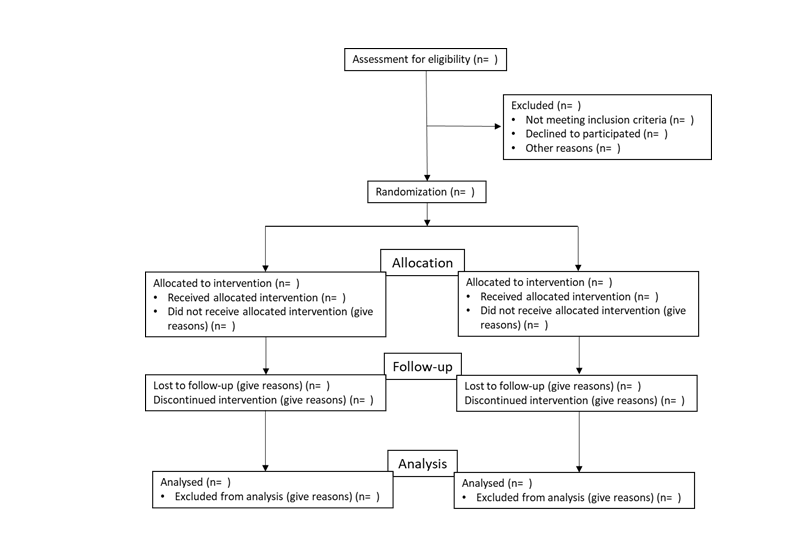

Supplement: S1 Fig — (TIF) [file pone.0342385.s001.tif]

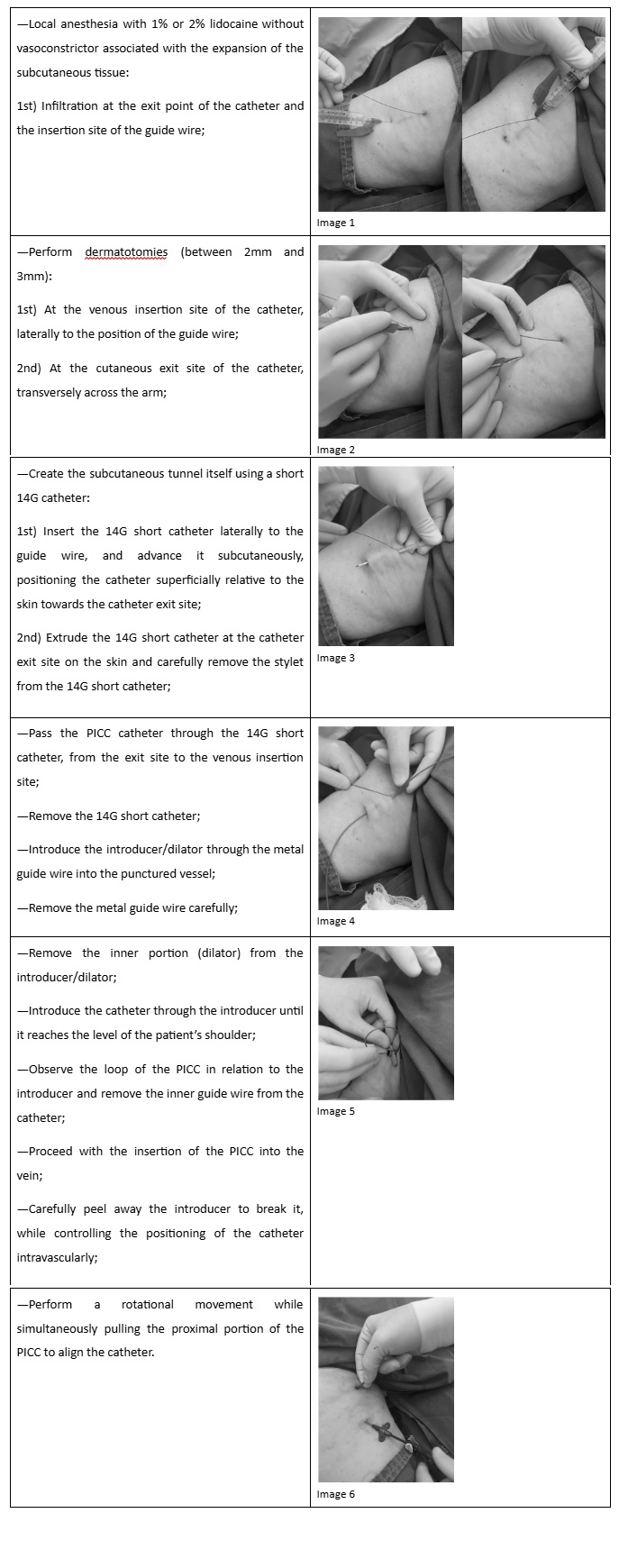

Supplement: S2 Fig — (TIF) [file pone.0342385.s002.tif]
