# Supplementary material for: Tunneled peripherally inserted central catheter versus non-tunneled and its effects in clinical outcomes: A multicenter randomized clinical trial protocol
Source: PLoS One. 2026 Mar 10;21(3):e0342385. doi: 10.1371/journal.pone.0342385 (PMC12974855; doi:10.1371/journal.pone.0342385)
Supplement: S3 Table — (PDF) [file pone.0342385.s003.pdf]

	STUDY PERIOD			
	Enrolment	Allocation	Post-allocation	Close-out
TIMEPOINT (Days)	*-1*	0	*1 to 30**	*After 30*
**ENROLMENT:**				
**Eligibility screen**	X			
**Informed consent**	X			
**Allocation**		X		
**INTERVENTIONS:**				
Tunneled PICC		X		
Non-tunneled PICC		X		
**ASSESSMENTS:**				
CLABSI, Thrombosis, Obstruction, Dislodgement		X		
PICC-related complications			X	
PICC dwell time free from complications			X	
Protocol of Tunneling				X

PICC: Peripherally Inserted Central Catheter
CLABSI: Central Line-Associated Bloodstream Infection
*Follow-up will be until 30 days after PICC insertion
