# Supplementary material for: Tunneled peripherally inserted central catheter versus non-tunneled and its effects in clinical outcomes: A multicenter randomized clinical trial protocol
Source: PLoS One. 2026 Mar 10;21(3):e0342385. doi: 10.1371/journal.pone.0342385 (PMC12974855; doi:10.1371/journal.pone.0342385)
Supplement: Attachment — Submitted filename: Reply letter.docx.pdf [file pone.0342385.s004.pdf]

Reply letter

December 17th, 2025.

PONE-D-25-38258

**TUNNELED PERIPHERALLY INSERTED CENTRAL CATHETER VERSUS NON-TUNNELED AND ITS EFFECTS IN CLINICAL OUTCOMES: A MULTICENTER RANDOMIZED CLINICAL TRIAL PROTOCOL**

Reviewer #1:

Dear Dr. Fabrizio D'Ascenzo, MD

Thank you for your review. We appreciate the feedback provided, which helped us improve the quality of our manuscript. The changes made in response have been highlighted in red, and we hope that they address all the concerns raised. Thank you for your consideration and we look forward to your response.

1) background in abstract should be shortened
   The background in abstract was shortened – Page 2

2) methods: it is not clear how randomization will be performed
   The randomization was better described in methods – Page 7

3) sample calculation is not clear to me>authors should state if they start from some infection rates and go back to others. Moreover it should be performed on primary end point
   Sample calculation was based on previous article, according to what is described in Sample size – Page 6
   HONG, Jiana; MAO, Xiaodan. Complications of tunneled and non-tunneled peripherally inserted central catheter placement in chemotherapy-treated cancer patients: a meta-analysis. Frontiers in Surgery, v. 11, p. 1469847, 2024.

4) do authors think that subgroup analysis should be performed
   We understand that a subgroup analysis could be performed, particularly with onco-hematologic patients. However, subgroup analysis will be conducted a posteriori.

Reviewer #2:

Dear Dr. Sandeep Tripathi, MS

Thank you for your review. We appreciate the feedback provided, which helped us improve the quality of our manuscript. The changes made in response have been highlighted in red, and we hope that they address all the concerns raised. Thank you for your consideration and we look forward to your response.

1) The authors' sample size is based on an estimate of a 50% reduction in composite outcomes. This appears very, very optimistic. The authors have not provided any information on how they estimated a 50% reduction. This is important because an inadequately powered study (because of overestimation of potential benefit) with standard frequentist analysis is very likely to lead to a null effect.
   Sample calculation was based on previous article, according to what is described in Sample size – Page 6;

HONG, Jiana; MAO, Xiaodan. Complications of tunneled and non-tunneled peripherally inserted central catheter placement in chemotherapy-treated cancer patients: a meta-analysis. Frontiers in Surgery, v. 11, p. 1469847, 2024.

2) The authors also mentioned that the research team would be blinded for tunneled vs. non-tunneled catheters. This is really not very feasible, as it is easy to identify tunneled vs. non-tunneled catheters. While blinding at the time of analysis can be ensured, data collection would have to be open-label, which is okay, as blinding is not realistic in this type of study.

   Thank you for your observation. However, the professionals responsible for assessing the outcomes will not be directly involved with the patients. The presence or absence of each outcome will be determined based on medical records.

3) The authors have also not mentioned any data collection on the success rate of inserting tunneled catheters and complications/costs associated with tunneled catheters compared to standard insertions. It seems like the authors wish to do an intention-to-treat analysis, which is okay. However, they should also do a per-protocol analysis as a sensitivity analysis.

   Thank you for your observation. All analyses will be performed following the intention-totreat principle. This is included in the analyses section.
